# Fetal Growth Restriction Impairs Lung Function and Neurodevelopment in an Early Preterm Rabbit Model

**DOI:** 10.3390/biomedicines11010139

**Published:** 2023-01-05

**Authors:** Ignacio Valenzuela, Katerina Zapletalova, Marnel Greyling, Yannick Regin, Andre Gie, David Basurto, Jan Deprest, Johannes van der Merwe

**Affiliations:** 1 Cluster Woman and Child, Department of Development and Regeneration, Group Biomedical Sciences, KU Leuven Herestraat 49, 3000 Leuven, Belgium; 2 Third Faculty of Medicine, Institute for the Care of Mother and Child, Charles University, 147 10 Prague, Czech Republic; 3 Department of Paediatrics and Child Health, Faculty of Medicine and Health Sciences, Stellenbosch University and Tygerberg Hospital, Cape Town 7505, South Africa; 4 Department of Obstetrics and Gynecology, Division Woman and Child, University Hospitals Leuven, Herestraat 49, 3000 Leuven, Belgium

**Keywords:** fetal growth restriction, prematurity, placental insufficiency, neurodevelopment, lung development, animal model, preclinical, rabbit

## Abstract

We previously reported the multi-system sequelae of fetal growth restriction, induced by placental underperfusion, in near-term born rabbits, in the immediate neonatal period and up to pre-adolescence. Herein, we describe the pulmonary and neurodevelopmental consequences of FGR in rabbits born preterm. We hypothesize that FGR has an additional detrimental effect on prematurity in both pulmonary function and neurodevelopment. FGR was induced at gestational day (GD) 25 by placental underperfusion, accomplished by partial uteroplacental vessel ligation in one uterine horn. Rabbits were delivered by cesarean section at GD 29, and placentas were harvested for histology. Neonates underwent neurobehavioral or pulmonary functional assessment at postnatal day 1, followed by brain or lung harvesting, respectively. The neurodevelopmental assessment included neurobehavioral testing and multiregional quantification of cell density and apoptosis in the brain. Lung assessment included functional testing, alveolar morphometry, and airway histology. FGR was associated with higher perinatal mortality, lower birth and placental weight, and a similar brain-to-body weight ratio compared to controls. Placental underperfusion decreased labyrinth and junction zone volumes in FGR placentas. FGR impaired pulmonary function, depicted by higher parenchymal resistance, damping, and elastance. Alveolar morphometry and airway smooth muscle content were comparable between groups. Neurobehavioral tests showed motoric and sensorial impairment in FGR rabbits. In FGR brains, cell density was globally reduced, with higher apoptosis in selected areas. In conclusion, in preterm-born rabbits, placental underperfusion leads to higher mortality, FGR, and impaired lung and brain development in early assessment. This study complements previous findings of placental, pulmonary, and neurodevelopmental impairment in near-term born rabbits in this model.

## 1. Introduction

Fetal growth restriction (FGR) is a leading cause of perinatal morbidity and mortality globally. It refers to the inability of a fetus to fulfill its genetic growth potential and has historically been defined by an estimated fetal weight under the 10th centile [1], although the addition of other diagnostic criteria has recently been suggested [2]. The incidence of FGR ranges between 3 and 24%, depending on multiple different population characteristics, being the highest in developing countries [3,4,5]. The most severe cases are usually those identified before 32 weeks gestation (early-onset FGR), with a perinatal mortality of almost 20% [6]. Moreover, survivors are at an increased risk of pulmonary, neurological, cardiovascular, and other neonatal complications, as well as mid- and long-term sequelae [7,8,9,10,11].

Currently, there is no prenatal treatment for FGR. The management is aimed at optimizing the timing of delivery wherein obstetricians must balance the risk of an ongoing pregnancy in the context of FGR against those of preterm delivery with subsequent prematurity [1,12]. Due to the severity of early-onset FGR, these fetuses are almost universally delivered preterm. Translational research could help identify novel prenatal interventions in the context of early FGR.

Animal models provide insight into the mechanisms behind FGR, allowing us to understand its sequelae better. Moreover, they are a necessary step before attempting clinical trials [13]. The rabbit has gained traction in the past decade as a preclinical model for perinatal research due to its similarities to humans [14]. Their organ and system development, along with their placental structure and physiology, resemble the human closer than most frequently used species, such as mice, rats, sheep, or pigs [15,16]. Specifically, the onset of lung alveolarization prior to birth [17], and their perinatal brain development [18], increase the translational value of rabbit models in preclinical research.

We characterized the early and late pulmonary and neurodevelopmental impairments of FGR induced by uteroplacental vessel ligation (UPVL) in rabbits born near term (gestational day 30; GD30) and the associated placental alterations resulting from placental underperfusion [19]. Since most severe FGR necessitates preterm delivery, it is essential to assess these sequelae in this context. To this point, we have shown that delivery at GD29 resembles the clinical scenario of early preterm birth in normally grown fetuses [20]. Herein, we characterize the placental and fetal consequences of FGR induced by UPVL in rabbits delivered at GD29.

## 2. Materials and Methods

### 2.1. Animal Model

Animals used in this project were treated according to current guidelines for animal well-being. All experiments were approved by the Ethics Committee for Animal Experimentation of the Faculty of Medicine (P080/2019) and are reported according to ARRIVE guidelines [21]. Time-mated rabbit dams (a hybrid of Dendermonde and New Zealand White) were housed in individual cages at 21 °C, 42% humidity, with a 12 h day/night cycle and free access to food and water. Conception day was considered day 0 of pregnancy. Fetal growth restriction was induced by uteroplacental vessel ligation, as previously reported [22]. Briefly, at gestational day (GD) 25 (full term 31.5 days), dams were administered induction anesthesia with IM ketamine (35 mg/kg Nimatek^®^, Eurovet Animal Health BV, Bladel, The Netherlands) and xylazine (5 mg/kg XYL-M^®^ 2%, VMD, Arendonk, Belgium), antibiotic prophylaxis (10 mg/kg enrofloxacin, Baytril^®^ 2.5% SC, Bayer, Diegem, Belgium), tocolysis (10 mg/kg medroxyprogesterone, Depo-Provera^®^ SC, Pfizer, Puurs, Belgium), and analgesia (0.03 mg/kg buprenorphine, Vetergesic^®^ SC, Ceva Animal Health, Brussels, Belgium) prior to surgery. Anesthesia was maintained with a continuous IV infusion of ketamine (8–16 mg/kg/h) and xylazine (2.4–4.8 mg/kg/h) while monitoring vital signs. Following laparotomy, 33–50% of the vessels going to each placenta were ligated in one random horn with Vicryl^®^ 5-0 (Ethicon^®^, Johnson & Johnson, Diegem, Belgium), leaving the contralateral horn as an internal control. The abdomen was closed with Vicryl^®^ 2-0 and Monocryl^®^ 3-0 (Ethicon^®^, Johnson & Johnson) for fascia and skin, respectively. The surgical wound was infiltrated with local anesthesia (2 mg/kg levobupivacaine, Chirocaine^®^, Abbvie, Wavre, Belgium) and sprayed with aluminum (Kela, Hoogstraten, Belgium).

Dams were monitored daily until delivery by caesarian section at GD 29. Following delivery, placentas were carefully separated from their implantation site, trimmed from the umbilical cord and membranes, blotted dry, weighed, and immerse-fixed in 4% paraformaldehyde (PFA) for 72 h. Dams were euthanized using IV phenytoin/pentobarbital (140 mg/kg Euthasol^®^, Kela). Kittens were pet dried, numbered, and kept in a warmed (34 °C) and humidified (55% RH) incubator. Later that day, they were stimulated to urinate, weighed, and fed a commercial milk substitute (Day One, protein 30%, fat 50%; Fox Valley) with added probiotics (Bio-Lapis; Probiotics International, Somerset, UK) and immunoglobulins (Col-o-Cat; SanoBest, Hertogenbosch, The Netherlands). At postnatal day 1 (PND1), litters with living cases and controls were allocated to either pulmonary or neurological assessment.

### 2.2. Placental Histology

After fixation, placentas were paraffin-embedded and cut into 4 um slides. Two slides per placenta were stained with cytokeratin lectin and scanned with the Zeiss AxioScan Z1 imaging platform (AxioScan Slide Scanner, Carl Zeiss MicroImaging GmbH, Munich, Germany). The detailed placental staining protocol can be found in Supplementary Information. Placental zones (decidua, labyrinth, junction zone) were manually delineated using the QuPath open-source software (version 0.2.0, Belfast, Northern Ireland) [23], and placental zone volumes were calculated from their relative volumes and placental weight, as previously described [19].

### 2.3. Pulmonary Function Testing (PFT)

On PND1, pressure–volume and forced oscillation maneuvers were performed using the FlexiVent system (SciReq; FlexiVent, Montreal, QC, Canada). After sedation with ketamine (35 mg/kg) and xylazine (6 mg/kg), a tracheostomy was performed, allowing the insertion of an 18-gauge metal cannula into the trachea. Rabbits were ventilated with a tidal volume of 10 mL/kg and positive end-expiratory pressure of 3 cmH_2_O at a rate of 120 breaths/min. In order to maximally inflate the lungs and standardize lung volume, two deep inflation maneuvers were performed prior to PFT until reaching a pressure of 30 cmH_2_O. Both pressure–volume (inspiratory capacity, static compliance, and static elastance) and forced oscillation tests (tissue damping, tissue elastance, central airway resistance, respiratory system resistance, dynamic compliance, and dynamic elastance) were performed as previously described [24]. The mean of 3 measurements for each maneuver, with a coefficient of determination > 95%, was calculated and used as a single data point for analysis.

### 2.4. Histological Lung Assessment

After PFT, the lungs were removed via thoracotomy, a 20-gauge catheter was fixed in the trachea, and the left lung was pressure fixed for 24 h at a constant hydrostatic pressure of 25 cmH_2_O in 4% PFA [24]. After PFA fixation, the left lung was paraffin-embedded and serially cut in 5 µm slides. Two slides per lung were stained with one of 2 protocols; (1) for alveolar morphometry, 1 slide per lung was stained with hematoxylin and eosin (H&E) and digitally scanned. Mean linear intercept (Lm), alveolar air space (Lma), and interalveolar septal thickness (Lmw) were calculated using a semi-automated, validated Fiji-plugin (ImageJ) (http://fiji.sc/Fiji; accessed on 1 October 2022) that randomly selected 20 fields per lung [25], according to stereological principles, as previously described [26]. (2) For airway smooth muscle content (ASMC), a primary α-smooth muscle actin (α-SMA) antibody (mouse anti-human, M0851; DakoCytomation, Glostrup, Denmark) was used in combination with a horseradish peroxidase-conjugated secondary antibody (goat anti-mouse, 115-035-044; Jackson ImmunoResearch, Ely, UK). Aminoethyl carbazole was used as a chromogen. Slides were digitally scanned, and ASMC was calculated on 10 randomly selected airways per lung. Airways with a diameter of 100–200 µm if cut in cross-section to their long axis were included. Airways with a long axis-to-short axis ratio of >2:1 were excluded. ASMC was analyzed in QuPath 0.2.0 [23] by manually selecting the fraction of stained muscle tissue and the full perimeter of the airway.

### 2.5. Neurobehavioral Assessment (NBA)

On PND1, kittens underwent a validated NBA protocol [27,28]. The short-term motor assessment comprised scoring of gait, posture, locomotion, head and limb activity, and activity duration [29,30]. Afterward, the cranial nerves, pain response, and righting reflex were tested for sensory evaluation [31]. All assessments were filmed and later scored by an observer blinded to the groups. The detailed NBA protocol can be found in Supplementary Information.

### 2.6. Brain Harvesting

Immediately after NBA, animals were deeply sedated with IM ketamine (35 mg/kg) and xylazine (6 mg/kg) and transcardially perfused with 0.9% saline + heparin (100 u/mL; 3 min at 30 mL/min) followed by 4% PFA (4 min at 33 mL/min). Brains were removed from the skull and further immersed and fixed in 4% PFA for 48 h.

### 2.7. Brain Histology

After fixation, brains were paraffin-embedded and serially sectioned at 4 µm. Three sets of four serial coronal sections every 100 µm were taken at each of the following two levels, as previously described [28]: level 1 started at the medial septal nucleus and level 2 at the hippocampal formation.

Six slides per brain (3 slides per level) were stained with Cresyl Violet (CV; C5042-10G; Sigma-Aldrich, Overijse, Belgium), and two slides per brain (one slide per level, eight slides in total) were incubated a terminal deoxynucleotidyl transferase dUTP nick end labeling (TUNEL) method for fluorescent in situ end labeling of double-stranded DNA fragmentation (Apoptag S7110; Millipore, Billerica, MA, USA). The secondary antibody was Alexa Fluor^®^ 488 goat anti-mouse conjugate (Sigma-Aldrich). Sections were counterstained with Hoechst 33342 (Sigma-Aldrich). Seven brain areas were assessed: frontal cortex, corpus callosum, caudate nucleus, internal capsule, putamen, hippocampus (dentate gyrus), and thalamus (anteroventral nucleus). The detailed acquisition and quantification methods can be found in Supplementary Information.

### 2.8. Statistical Analysis

Data were analyzed using GraphPad Prism version 9.0.0 for MacOS (GraphPad Software, San Diego, CA, USA, www.graphpad.com) and RStudio (Rstudio: Integrated Development for R. Rstudio, PBC, Boston, MA, USA). Data distribution was checked for normality and presented as mean with standard deviation or median with interquartile range, as appropriate. Data comparison was made by Fisher’s exact, or a linear mixed-effects model, considering the mother (litter) as a random effect and the group (FGR or control) as a fixed effect. A *p*-value of <0.05 was considered significant.

## 3. Results

### 3.1. UPVL Induced Placental Alterations Leading to Fetal Growth Restriction and Increased Perinatal Mortality at GD29

Eighteen does delivered sixty-three live kittens from the ligated horns and eighty-six from the control horns. UPVL resulted in higher fetal (*p* < 0.0001) and neonatal mortality (*p* < 0.0001). Four litters had no FGR survivors at PND1 and were excluded from further assessment. Kittens from the ligated horn had lower birth weight (*p* < 0.0001), placental weight (*p* = 0.008), fetal-to-placental weight ratio (*p* = 0.008), and brain weight (*p* = 0.0001). Brain-to-body weight ratio did not differ between groups (Table 1).

**Figure 1:**
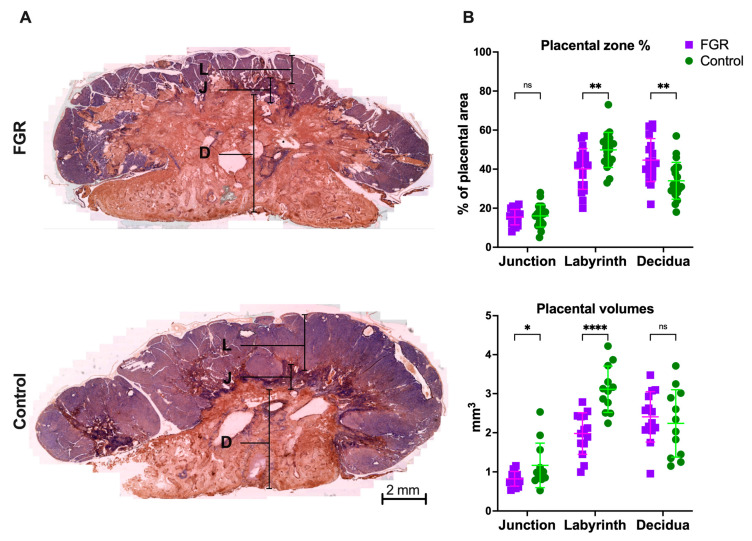
Placental histology: (**A**) placentas with cytokeratin/lectin double staining; (**B**) placental zone proportions from 19 FGR and 20 control placentas from 13 litters. Data were analyzed using a linear mixed-effects model and displayed as median ± IQR with significance as *ns p* > 0.05; * 0.05 ≥ *p* > 0.01; ** 0.01 ≥ *p* > 0.001.; **** *p* < 0.0001. D: decidua; FGR: fetal growth restriction; J: junction zone; L: labyrinth zone.

Additionally, placentas from FGR kittens had reduced labyrinth (3.17 ± 0.5 vs. 2.03 ± 0.5 mm^3^; *p* < 0.0001) and junction zone volumes (1.23 ± 0.5 vs. 0.79 ± 0.1 mm^3^; *p* = 0.013), while decidual volumes were similar between groups (2.24 ± 0.8 vs. 2.07 ± 0.6; *p* = 0.5; Figure 1). Proportional to total placental volume, the labyrinth zone was reduced (40.1 ± 10 vs. 49.8 ± 8%; *p* = 0.003), and the decidua was increased (44.6 ± 11 vs. 34.1 ± 9%; *p* = 0.002) in FGR placentas, while the junction zone was proportionally comparable between groups (15.4 ± 3 vs. 16.1 ± 5%; *p* = 0.7).

### 3.2. FGR Lungs Had Increased Peripheral Tissue Damping and Resistance, but Similar Airway Mechanics

**Figure 2:**
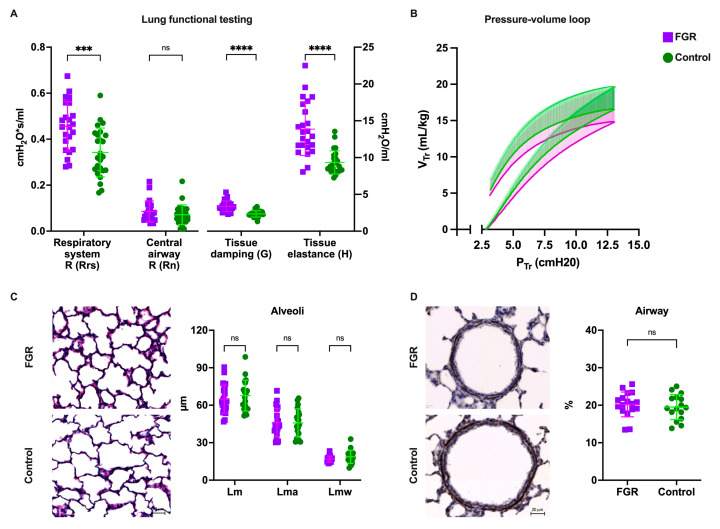
Pulmonary assessment tests at postnatal day 1: (**A**) biomechanical properties of parenchymal tissue and central airway; (**B**) pressure–volume curve, corrected by body weight; (**C**) alveolar morphometry in H&E slides; (**D**) airway smooth muscle content in α-SMA slides. Data were analyzed using a linear mixed-effects model and displayed as mean ± SD (**A**,**C**) or median and IQR (**D**), with significance as *ns p* >0.05; *** 0.001 > *p* > 0.0001; **** *p* < 0.0001. FGR: fetal growth restriction; Lm: mean linear intercept; Lma: alveolar air space; Lmw: interalveolar septal thickness; R: resistance.

Lung function was significantly reduced in FGR animals. Forced oscillation tests showed increased tissue damping (3.35 ± 0.7 vs. 2.33 ± 0.4 cmH_2_O/mL; *p* < 0.0001), tissue elastance (13.8 ± 3.6 vs. 9.31 ± 1.5 cmH_2_O/mL; *p* < 0.0001), and respiratory system resistance (0.465 ± 0.1 vs. 0.335 ± 0.1 cmH_2_O•s/mL; *p* < 0.0001) in FGR lungs, while the large conducting airway resistance was not significantly different between groups (Figure 2A). In pressure–volume perturbations, FGR lungs had significantly reduced hysteresis (1.03 ± 0.1 vs. 1.28 ± 0.1 mL•cmH_2_O; *p* = 0.01), while static compliance was not different after correcting to body weight (Figure 2B).

### 3.3. Alveolar Morphometry Is Comparable in Premature Lungs from FGR and Normally Grown Subjects

Both groups had similar lung sizes when corrected to body weight, as measured by inspiratory capacity (26.90 ± 4.42 vs. 27.94 ± 6.24 mL/g; *p* = 0.5). On histological assessment, alveolar size (Lm; 68.67 ± 3.01 vs. 70.78 ± 4.33 µm; *p* = 0.4), alveolar airspace (Lma; 51.64 ± 2.28 vs. 50.59 ± 5.22 µm; *p* = 0.6), and alveolar wall thickness (Lmw; 18.31 ± 3.02 vs. 21.37 ± 2.53 µm; *p* = 0.3) was similar between groups (Figure 2C). On airway assessment, ASMC was also similar between groups (18.1 ± 3 vs. 18.8 ± 3%; *p* = 0.4; Figure 2D).

### 3.4. Neurobehavioral Impairment Coincides with Globally Reduced Cell Density in FGR Brains

**Figure 3:**
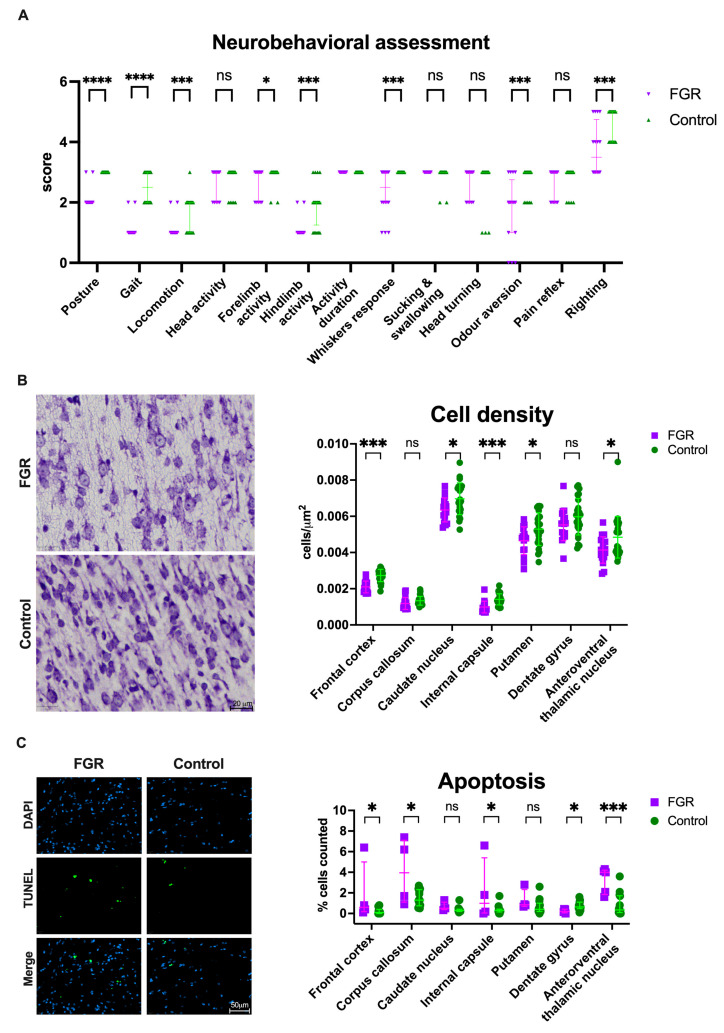
Neuropathology in postnatal day 1 rabbit brains: (**A**) Neurobehavioral tests grouped scores from 18 FGR and 25 control subjects from 7 litters. (**B**) Cell density was assessed in cresyl violet stained slides. Representative images on the left depict cell density in the frontal cortex of FGR and control brains. To the right, cell density from 17 FGR and 25 control subjects from 7 litters. (**C**) Representative images on the left show apoptosis (TUNEL+ cells) in corpus callosum. Data from 5 FGR and 15 control subjects from 5 litters. Data were analyzed using a linear mixed-effects model and displayed as mean ± SD (**A**) or median and IQR (**B**) with significance as *ns p* >0.05; * 0.05 ≥ *p* > 0.01; *** 0.001 > *p* > 0.0001; **** *p* < 0.0001. FGR: fetal growth restriction.

FGR was associated with neurodevelopmental impairment, as shown by lower motoric and sensorial scores on neurobehavioral assessment (Figure 3A). FGR rabbits had abnormal posture, gait, locomotion, cranial nerve activity, and righting reflex. Brains from FGR newborns had reduced cell density in the frontal cortex (*p* < 0.0001), caudate nucleus (*p* = 0.02), internal capsule (*p* = 0.0001), putamen (*p* = 0.03), and thalamus (*p* = 0.03; Figure 3B) and similar density in the corpus callosum (*p* = 0.067) and dentate gyrus (*p* = 0.11). Moreover, apoptosis was increased in the frontal cortex (*p* = 0.03), corpus callosum (*p* = 0.01), internal capsule (*p* = 0.03), and thalamus (*p* = 0.0001) and reduced in the dentate gyrus (*p* = 0.01; Figure 3C). The detailed neuropathological results can be found in Supplementary Information, Table S1.

## 4. Discussion

In rabbits born preterm (GD29), FGR leads to multiorgan impairment, herein depicted by impaired pulmonary function, neurobehavioral deficit, and globally reduced cell density in the brain. These results demonstrate the detrimental effects of placental underperfusion at an earlier time point than previously studied [19]. Despite a shorter exposure to placental underperfusion, FGR rabbits delivered preterm had significantly worse outcomes than their normally grown premature siblings.

Consistent with previous reports from this model, the forced oscillation tests demonstrate disrupted biomechanical properties in the FGR peripheral lung compartment. On the other hand, there were no significant differences in alveolar morphology. This suggests that the peripheral (i.e., parenchymal) effect of FGR is not uniquely alveolar. Considering that alveolarization is not fully established at this gestational age in the rabbit lung—nor in the equivalent human scenario—alveolar morphometric differences are likely missed by assessing at this time point since the effect is more pronounced during the rapid fetal growth and alveolar development towards the end of gestation.

Most early-onset FGR cases are delivered during the saccular stage of lung development, before the onset of alveolarization, which in humans takes place at 36 weeks, and in rabbits at GD30 [32,33]. Moreover, the smooth muscle was predictably similar between groups as the changes in the large conducting airway are likely related to postnatal exposure/injury [34,35,36].

On neurodevelopmental assessment, neurobehavioral impairment coincided with neuropathological findings in premature FGR neonates. This neurobehavioral deficit has been previously characterized up to pre-adolescence and relates to anxiety-like behavior and memory impairment in the long-term assessment [37,38,39]. Additionally, consistent with previous reports, an insult at GD25 impacts both grey and white matter structures [40]; at that point, pre-oligodendrocyte expansion is accompanied by a progressive increase in immature oligodendrocytes [40]. Several studies have shown this to be a highly vulnerable period for brain development [40,41,42]. Since fetuses presenting early onset FGR are delivered between 28 and 37 weeks, characterizing the rabbit brain in this context is evidently relevant.

We previously described the microvascular placental effects of UPVL in this model in GD30 placentas [19]. In this study, we observed that the marked effect in the labyrinth zone is already detectable at GD29. Placental alterations found in this model coincide with the higher incidence of placental lesions found in human placentas from early-onset FGR pregnancies in comparison to late-onset [43,44,45].

We acknowledge that our study has limitations. The terminal nature of our tests did not allow for long-term or longitudinal examination of lung or brain development. Although it might be difficult to accomplish given the high perinatal mortality of the model at this time point, assessment after the neonatal period would provide a much broader understanding of the long-term effects of FGR. Additionally, we did not investigate the underperfusion effects in all organs but only selected key organ systems known to be affected by FGR. In order to assess the true potential of potential therapies, future studies need to characterize the effects on other major organ systems.

## 5. Conclusions

We expanded the characterization of this suitable model in a relevant time point and confirmed that the detrimental pulmonary and neurodevelopmental effects of placental underperfusion are significant despite a shorter exposure. Moreover, the expected morbidity of premature controls did not mask these multiorgan impairments. These sequelae, along with the previously described, can be used as targets in the development of prenatal treatments or interventions in translational research.

## Figures and Tables

**Table 1:** Survival and biometrics.

N = 18 Dams	FGR	Control	*p*-Value
Survival at birth	63/114 (55.3%)	86/89 (96.6%)	<0.0001
Survival at PND1	46/114 (40.3%)	77/89 (86.5%)	<0.0001
Birth weight (g)	32.09 ± 6.15	40.01 ± 5.94	<0.0001
Placental weight (g)	5.14 ± 1.19	5.78 ± 1.10	0.008
Brain weight (g)	1.53 ± 0.19	1.72 ± 0.19	0.0001
Brain/body weight ratio	0.048 ± 0.008	0.045 ± 0.006	0.09
Fetal/placental weight ratio	6.40 ± 1.11	7.08 ± 1.20	0.008

Survival and birth weight were assessed 4 h after caesarian delivery. Brain weight was measured at PND1. Data expressed as n (%) or mean ± SD. FGR: fetal growth restriction; PND1: postnatal day 1.

## Data Availability

The datasets analyzed during the current study are available from the corresponding author upon reasonable request.

## Supplementary Materials

The following supporting information can be downloaded at: https://www.mdpi.com/article/10.3390/biomedicines11010139/s1, Table S1: Neuropathological assessment in postnatal day 1 brains.
