# Supplementary material for: Fetal Growth Restriction Impairs Lung Function and Neurodevelopment in an Early Preterm Rabbit Model
Source: Biomedicines. 2023 Jan 5;11(1):139. doi: 10.3390/biomedicines11010139 (PMC9855731; doi:10.3390/biomedicines11010139)
Supplement: Supplementary file 1 [file biomedicines-11-00139-s001.zip › biomedicines-2113843-supplementary.pdf]

SUPPLEMENTARY INFORMATION

Fetal growth restriction impairs lung function and neurodevelopment in an early preterm rabbit model.

Ignacio Valenzuela, Katerina Zapletalova, Marnel Greyling, Yannick Regin, Andre Gie, David Basurto, Jan Deprest, Johannes van der Merwe.

Materials and methods

Placental immunohistochemistry

Sections were dewaxed and rehydrated to tris-buffered saline (TBS, which was used for all wash steps further in the protocol). Antigen retrieval was performed using Pepsin (Sigma, 0.04% in preheated 0.01 M HCl, 10min, 37 °C). Slides were washed in TBS (once at 4 °C and then twice at RT). Non-specific binding was blocked using blocking buffer (dH_2O containing 2% bovine serum albumin, 1% skimmed dry milk and 0.1% Tween20, 15min, 3.3% Normal Goat Serum, RT). Sections were then incubated with Rabbit anti-Cytokeratin MNF116 (Agilent M0821;1:200 (0.8µg/ml); 2h 37°C). Bound antibody was detected with Goat anti-Mouse (Abcam; ab102445; 1:25; 30 min.) attached with APAAP Complex (STAR67; Bio-Rad; 1:50), which converts Fast Blue BB (F3378; Sigma) into a blue product. Sections were washed in TBS and then endogenous peroxidase activity was blocked with hydrogen peroxide (3% in methanol) for 30min at room temperature (RT). Sections were washed and then incubated with blocking buffer containing 10% goat serum for 15 min at RT. Slides were incubated with biotinylated lectin (isolectin B4, B-1205, Vector Laboratories; 1:100, 90min, 37 °C) before detection with horseradish peroxide-conjugated streptavidin (P0397, Agilent; 1:840, 30 min, RT) followed by 3′-*Diaminobenzidine* (Sigma, 20 min RT). Slides were washed in water and counterstained with Nuclear Fast Red (Vector laboratories, 5min) before dehydrating and mounting with Neo-Clear.

Neurobehavioral assessment

Neurobehavioral PND1 evaluation based on a modification of neurobehavioral scoring protocol previously described[1]. For each animal, testing is videotaped and scored by a blinded observer. The kittens are evaluated in a designated space close to their pen with auditory and olfactory contamination kept to a minimum. Before handling they remain undisturbed in this assessment area for a 3-5min adaptation period.

- Cranial nerves are assessed by testing smell (olfaction is tested by recording time to aversive response to a cotton swab soaked with pure ethanol), sucking, and swallowing (by introduction of formula into the kittens' mouth with a plastic syringe), and head turn to feeding. The responses are graded on a scale of 0 to 3, 0 being the worst response and 3 the best response.
- Motor examination includes tone, motor activity, and locomotion on a flat surface, righting reflex, and gait. The righting reflex is assessed when the kittens are placed on their backs and the number of times turned prone (within 2s) from supine position in 5 tries is registered. Gait

is examined based on a modification by Georgiadis et al.[2] Locomotion is assessed as described by Kannan et al.[3]

- Sensory examination is limited to touch on the whiskers with a brush on both sides as well as pain on limbs (mild pin prick).

Neuropathology: Image acquisition and quantification

Histological slides were digitized using the Zeiss AxioScan Z1 imaging platform (AxioScan Slide Scanner, Carl Zeiss MicroImaging GmbH), using a 20x Plan Apochromat objective coupled to a 3 Chip CCD Camera (Hamamatsu Photonics, Japan). All focusing and field-of-view assembly was done by the integrated Carl Zeiss Zen software.

Quantification of cell density and immunohistochemistry-positive cells was done according to previous reports by our group[4]. Briefly, for cell quantification in the frontal cortex, regions of interest (ROI) were selected on 3 consecutive slides per level separated by 100 µm. In each ROI, 5 squares (100 x 100-µm) were selected at low magnification so individual cells were not visible to avoid bias. Cells were manually counted in these squares, and cell density was calculated dividing the total number of cells by the area. For all other ROI, the cell detection tool from QuPath was utilized to measure cell density.

For quantification of the immunohistochemistry-positive cells in the TUNEL stains quantification profiles on the digitized whole-slide images were obtained with QuPath software[5]. Herein the whole ROI was delineated, and quantification was done by using the fast cell counting and positive cell detection functions. Positive cell counts were expressed as percentage of positive cells per total cells detected.

Table S1. Neuropathological assessment in postnatal day 1 brains.

Parameter	FGR	Control	p-value
**Neuron density, cells/μm^2**			
Frontal cortex	0.0021 ± 0.0003	0.0027 ± 0.0003	<0.0001
Corpus callosum	0.0012 ± 0.0003	0.0014 ± 0.0002	0.067
Caudate nucleus	0.0070 ± 0.0007	0.0063 ± 0.0009	0.021
Putamen	0.0047 ± 0.0003	0.0053 ± 0.0003	0.034
Internal capsule	0.0010 ± 0.0008	0.0014 ± 0.0009	0.0002
Hippocampus DG	0.0055 ± 0.0009	0.0060 ± 0.0010	0.11
AVTN	0.0043 ± 0.0007	0.0049 ± 0.0011	0.030
**TUNEL, % of positive cells**			
Frontal cortex	0.60 (4.8)	0.09 (0.4)	0.036
Corpus callosum	3.95 (5.9)	1.30 (1.57)	0.011
Caudate nucleus	0.50 (0.7)	0.30 (0.21)	0.082
Internal capsule	0.99 (5.3)	0.30 (0.27)	0.038
Putamen	0.85 (1.63)	0.40 (0.8)	0.11
Hippocampus DG	0.20 (0.35)	0.63 (0.63)	0.018
AVTN	4.0 (2.35)	0.35 (1.63)	0.0001

Cell density from 17 FGR and 25 control subjects from 7 litters. Expression of TUNEL was measured in 5 FGR and 15 control brains from 5 litters. Data were analysed using a linear mixed-effects model, displayed as mean ± SD or median (IQR). AVTN, anteroventral thalamic nucleus; DG, dentate gyrus.

References

1 Derrick, M. *et al.* Preterm fetal hypoxia-ischemia causes hypertonia and motor deficits in the neonatal rabbit: a model for human cerebral palsy? *J Neurosci* **24**, 24-34, doi:10.1523/JNEUROSCI.2816-03.2004 (2004).

2 Georgiadis, P. *et al.* Characterization of acute brain injuries and neurobehavioral profiles in a rabbit model of germinal matrix hemorrhage. *Stroke* **39**, 3378-3388, doi:10.1161/STROKEAHA.107.510883 (2008).

3 Kannan, S. *et al.* Dendrimer-based postnatal therapy for neuroinflammation and cerebral palsy in a rabbit model. *Sci Transl Med* **4**, 130ra146, doi:10.1126/scitranslmed.3003162 (2012).

4 Van der Veeken, L. *et al.* Maternal surgery during pregnancy has a transient adverse effect on the developing fetal rabbit brain. *American journal of obstetrics and gynecology* **221**, 355 e351-355 e319, doi:https://doi.org/10.1016/j.ajog.2019.07.029 (2019).

5 Bankhead, P. *et al.* QuPath: Open source software for digital pathology image analysis. *Scientific reports* **7**, 16878, doi:10.1038/s41598-017-17204-5 (2017).
